# Supplementary material for: Splice-Junction-Based Mapping of Alternative Isoforms in the Human Proteome
Source: Cell Rep. Author manuscript; Available in PMC 2020 Jan 15. (PMC6961840; doi:10.1016/j.celrep.2019.11.026)
Supplement: 2 [file NIHMS1546469-supplement-2.zip › DF1/PXD009737/TestisLysC/TestisLysC_32_SMU1_REALKWQQHQGLLPPGMTIDLFRGK.pdf]

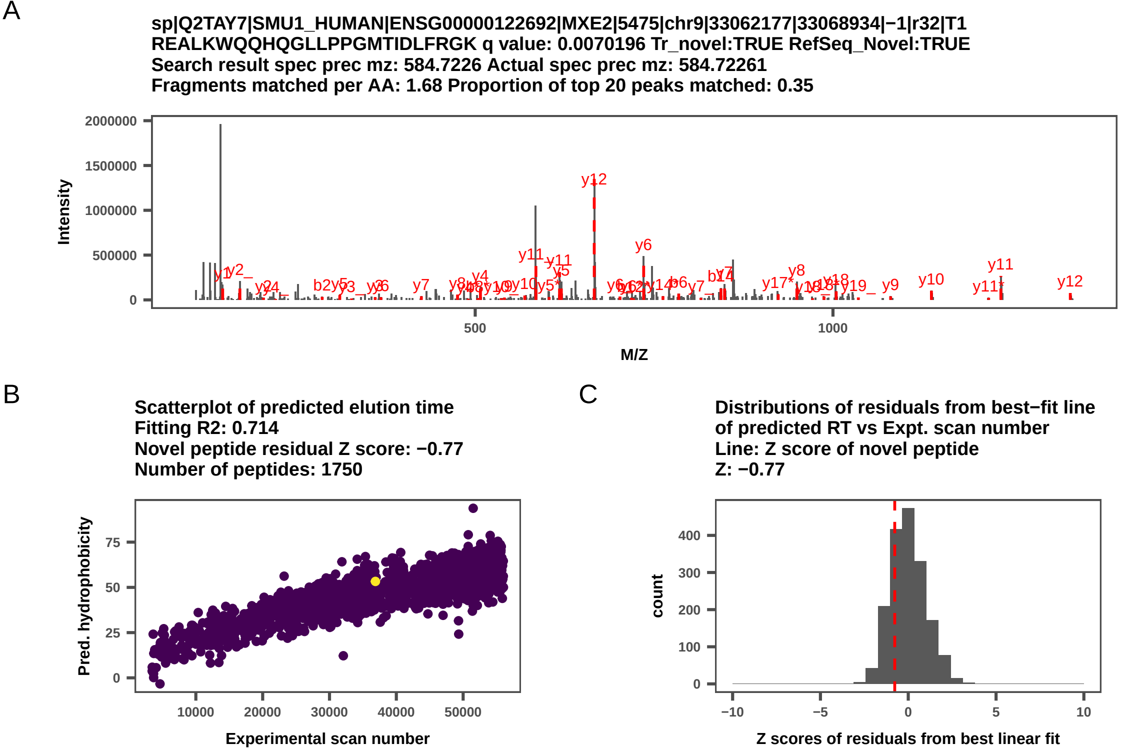
A
sp|Q2TAY7|SMU1_HUMAN|ENSG00000122692|MXE2|5475|chr9|33062177|33068934|−1|r32|T1
REALKWQQHQGLLPPGMTIDLFRGK q value: 0.0070196 Tr_novel:TRUE RefSeq_Novel:TRUE
Search result spec prec mz: 584.7226 Actual spec prec mz: 584.72261
Fragments matched per AA: 1.68 Proportion of top 20 peaks matched: 0.35
Intensity
2000000
1500000
1000000
500000
0
y12
y6
y11
y5
y8
y10
y11
y12
y9
y7
500
1000
M/Z
B
Scatterplot of predicted elution time
Fitting R2: 0.714
Novel peptide residual Z score: −0.77
Number of peptides: 1750
Pred. hydrophobicity
75
50
25
0
10000
20000
30000
40000
50000
Experimental scan number
C
Distributions of residuals from best−fit line
of predicted RT vs Expt. scan number
Line: Z score of novel peptide
Z: −0.77
count
400
300
200
100
0
−10
−5
0
5
10
Z scores of residuals from best linear fit
